# Shock, Cardiac Arrest, and Resuscitation

**DOI:** 10.1155/2017/5743702

**Published:** 2017-01-03

**Authors:** Yan-Ren Lin, Kee-Chong Ng, Aristomenis K. Exadaktylos, John M. Ryan, Han-Ping Wu

**Affiliations:** ^1^Department of Emergency Medicine, Changhua Christian Hospital, Changhua, Taiwan; ^2^School of Medicine, Kaohsiung Medical University, Kaohsiung, Taiwan; ^3^School of Medicine, Chung Shan Medical University, Taichung, Taiwan; ^4^Department of Emergency Medicine, KK Women's and Children's Hospital, Singapore; ^5^Department of Emergency Medicine, Bern University Hospital, Freiburgstrasse, Bern, Switzerland; ^6^Emergency Department, St Vincent's University Hospital, Elm Park, Dublin, Ireland; ^7^Division of Pediatric General Medicine, Department of Pediatrics, Chang Gung Memorial Hospital at Linko, Kweishan, Taoyuan, Taiwan; ^8^College of Medicine, Chang Gung University, Taoyuan, Taiwan

The required knowledge regarding basic life support (BLS), advanced cardiovascular life support (ACLS), and postresuscitative care in the United States and Europe was updated by the American Heart Association (AHA) and European Resuscitation Council (ERC), respectively, in 2015 [1–3]. These new updates have guided the global treatment strategies of critical and emergency care [1–3]. However, in spite of these new guidelines, some studies have reported that the survival rates and neurological outcomes of patients experiencing out-of-hospital cardiac arrest (OHCA) were still not obviously improved in recent years [4–6]. Resuscitating a patient with OHCA is still a challenge for primary physicians; therefore, better treatment strategies are necessary. Novel categories that focus on postresuscitative care, resuscitation, or shock management are still of interest to scientific researchers. To improve patient outcomes, current guidelines, and knowledge can be clinically applied or even challenged. In this special issue, we would like to provide an opportunity to widely introduce related works discussing shock, cardiac arrest, and resuscitation.

First, topics related to cardiopulmonary resuscitation (CPR) training and quality control were described in detail. S. R. Gauna et al. introduced and discussed a system that provided accurate feedback on the chest compression depth and rate on soft surfaces. Their solution compensated for mattress displacement and avoided overestimation of the compression depth. In addition, C. Ahn et al. evaluated smartphone applications for CPR training in South Korea. Their study suggested that smartphone-based CPR training apps should include accurate CPR information and be easy to use for any layperson who could be a potential rescuer in real-world incidents. J. H. Lee et al. discussed the effect of the duration of BLS training on the students' subsequent cardiopulmonary and automated external defibrillator skills. They concluded that the retention of high-quality CPR skills required longer and more hands-on training, particularly with automated external defibrillators (AEDs). S. Calicchia et al. reported a training experience on a BLSD (Basic Life Support and Defibrillation) module designed for a group of pupils in an Italian primary school. They found that life-saving maneuvers can be effectively taught to primary school students. M. Sadrawi et al. focused on analyzing the quality of CPR using a filtered raw ECG signal. The results showed that patients younger than 60 years of age with a higher complexity of CPR-intrinsic mode functions and increased amplitude differences have a higher survival rate. W. W. D. Yong et al. also analyzed the quality of CPR training. In their study, the pedagogy recommendations for trainers of dispatcher-assisted CPR programs were developed using the Delphi method. A. Khoury et al. evaluated the factors that might influence the performance of bag-valve-mask ventilation. Y.-J. Syue et al. considered the prognosis of patients who experienced in-hospital cardiac arrests of cardiac and noncardiac origin during night shifts. They found that in-hospital cardiac arrests (IHCAs) that occur at night correspond to an increased mortality, which was more apparent for IHCAs of cardiac origin than for those of noncardiac origin.

Second, this issue includes an animal model of ventricular fibrillation induced cardiac arrest reported by G. K. Venkata et al. Their study concluded that postarrest myocardial dysfunction resulted in segmental wall motion defects primarily in the left anterior descending coronary artery region. Furthermore, there were no perfusion defects in the involved segments. In addition, there are two articles discussing cardiovascular disease and biomarkers for predicting sepsis. C.-M. Lin et al. found that a “resistance index” from a carotid ultrasound could detect flow changes before and after the stenting procedure, thus having a great capacity to replace the role of the computed tomography perfusion exam. M.-Y. Huang et al. noted that dynamic changes in the procalcitonin and procalcitonin clearance could serve as a predictor of survival in critically ill patients with severe sepsis. Finally, in this special issue, we have an excellent review article focused on hypotensive resuscitation among trauma patients. The authors (M. M. C. et al.) mentioned that lowering target blood pressures in trauma patients might be more beneficial than highly aggressive fluid resuscitation in prehospital and hospital settings. However, this new concept still requires more trials or evidence to clarify this result. Finally, this special issue highlights several articles that report improvements in CPR training, quality control, resuscitation strategies, and sepsis prediction. We believe that readers could obtain useful information from this issue.

*Yan-Ren Lin* *Kee-Chong Ng* *Aristomenis K. Exadaktylos* *John M. Ryan* *Han-Ping Wu*
